# Anti-SARS-CoV-2 Vaccination and PIMS-TS—Friends or Foe? Case Reports and Literature Review

**DOI:** 10.3390/vaccines12030278

**Published:** 2024-03-07

**Authors:** Violetta Opoka-Winiarska, Izabela Morawska-Michalska, Paulina Mertowska, Krzysztof Gosik, Olga Kądziołka, Ewelina Grywalska

**Affiliations:** 1 Department of Pediatric Pulmonology and Rheumatology, Medical University of Lublin, 20-093 Lublin, Poland; violetta.opoka-winiarska@umlub.pl; 2 Department of Clinical Immunology, Medical University of Lublin, 20-093 Lublin, Poland; izabelamorawska@gmail.com; 3 Department of Experimental Immunology, Medical University of Lublin, 20-093 Lublin, Poland; krzysztof.gosik@umlub.pl (K.G.); ewelina.grywalska@umlub.pl (E.G.); 4 Department of Paediatric Pulmonology and Rheumatology, University Children’s Hospital of Lublin, 20-093 Lublin, Poland; olga.kadziolka@uszd.lublin.pl

**Keywords:** COVID-19, COVID-19 vaccination, MIS-C, PIMS-TS, SARS-CoV-2

## Abstract

Pediatric inflammatory, multisystem syndrome temporally associated with SARS-CoV-2 infection (PIMS-TS), also known as a multisystem inflammatory syndrome in children (MIS-C), is diagnosed in children who develop an inadequate inflammatory response after exposure to the SARS-CoV-2 virus. The pathogenesis of the abnormal response of the immune system to a previous SARS-COV-2 infection has not been explained. Similarly, the safety and effectiveness of COVID-19 vaccinations in this group of patients have become the subject of clinical discussion. Presenting experiences from many centers aims to answer this question. We present 4 cases of patients who suffered from PIMS-TS. Three of them were safely vaccinated against COVID-19 after illness. One patient developed PIMS-TS temporarily associated with COVID-19 vaccination. We also collected and discussed data from other centers.

## 1. Introduction

Pediatric inflammatory, multisystem syndrome temporally associated with SARS-CoV-2 infection (PIMS-TS), also known as a multisystem inflammatory syndrome in children (MIS-C), is diagnosed in children who develop an inadequate inflammatory response after exposure to the SARS-CoV-2 virus [1]. The pathogenesis of this abnormal response to viral antigens is not entirely clear; however, it is postulated that many immunological mechanisms and responses to the so-called superantigen take part in hyperinflammation [2,3,4]. The symptoms of PIMS-TS resemble Kawasaki disease (KD), systemic juvenile idiopathic arthritis (JIA), and macrophage activation syndrome (MAS), and they require thorough investigation to be properly differentiated. Typically, children have a high fever (over 38.5 degrees Celsius), abdominal pain, vomits, conjunctivitis, oral mucosa inflammation, skin rash, as well as respiratory and cardiac symptoms up to pericardial effusion and symptoms of shock. In laboratory tests, attention is drawn to high inflammatory markers ESR, CRP, procalcitonin, D-dimers, and often elevated levels of cardiac troponins [5]. Those parameters quickly normalize after proper treatment is started. 

Treatment includes intravenous infusions of immunoglobulins (IVIG), acetylsalicylic acid (ASA), and, if necessary, systemic glucocorticosteroids (GCS), as well as immunomodulating drugs such as anti-IL-15. Some children require intensive care as PIMS-TS can be life-threatening, and permanent complications may also develop [6]. Scientific evidence indicates that vaccination against COVID-19 is safe and effective in this group of children [7,8,9]. However, the question remains ambiguous—can patients with a history of PIMS-TS be safely vaccinated, and whether the development of PIMS-TS can be triggered by the COVID-19 vaccine? This publication aims to present four clinical cases regarding PIMS-TS in children and to analyze the available literature on this extremely important issue. The cases presented in this publication of patients who developed PIMS-TS both before and after receiving the COVID-19 vaccine allow for comparative analysis to enrich our knowledge of the pathogenesis of this syndrome, highlighting potential differences in triggering factors and mechanisms between cases caused by infection and cases potentially related to vaccination. This detailed understanding is critical to the ongoing safety assessment of the COVID-19 vaccine, particularly in pediatric populations where the risk and impact of PIMS-TS are important. From a clinical perspective, understanding the full spectrum of PIMS-TS symptoms, whether they occur before or after vaccination, is invaluable to healthcare providers. It equips them with the knowledge to better recognize, manage, and treat this complex syndrome, ensuring timely and effective care for affected patients.

Additionally, transparent reporting and discussion of PIMS-TS cases, including rare cases occurring after vaccination, plays a key role in building and maintaining public trust in the vaccination process. It emphasizes a commitment to safety and transparency, key elements of public health communication that encourage informed decision-making and vaccine acceptance.

## 2. Materials and Methods

### 2.1. Patient Characteristics

In our study, we present 3 cases of patients diagnosed with PIMS-TS who, after total recovery, were vaccinated with the anti-SARS-CoV-2 Pfizer BioNTech vaccine. We also present one case of a boy who developed PIMS-TS in a temporal relationship with the anti-SARS-CoV-2 Pfizer BioNTech vaccine (1. dose). Detailed information about the described patients is provided in Table 1. All children were vaccinated following the national vaccination program until the disease occurred without any post-vaccination side effects. None of the children had any previous symptoms or severe infections indicating immunodeficiency. All patients are still under observation (currently every 12 months) with no symptoms of chronic disease. Patient classification as PIMS-TS cases was developed according to the CDC case definition [10].

### 2.2. Literature Review Strategy

We also decided to review the available literature on this topic. The first aspect addressed in the literature review is the safety and effectiveness of vaccination in children who have undergone PIMS-TS in the past. In the second part, we focus on the issue of PIMS-TS in previously vaccinated children. For this purpose, we analyzed the available literature using the Pubmed, Scopus, and Google Scholar databases. To find the data as efficiently as possible, we used the MeSH thesaurus with the terms COVID-19 vaccines (all synonyms) and PIMS-TS (all synonyms). 

## 3. Results

### 3.1. Case 1

The first patient is a 6-year-old female admitted to the Department of Pediatric Rheumatology and diagnosed with PIMS-TS according to the appropriate criteria in December 2020. Three weeks earlier, the child had contact with a kindergarten teacher who was sick with COVID-19. Apart from fever, the symptoms included macular rash, pharyngitis, pericardial effusion, and coagulation abnormalities. Laboratory tests showed hyperferritinemia, hyponatremia, high erythrocyte sedimentation rate (ESR) and C-reactive protein (CRP), prolonged clotting times, and high titers of anti-SARS-CoV-2 IgG antibodies. SARS- SARS-CoV-2 PCR test was negative.

She was treated with IVIG, GKS, and ASA. She was discharged from the hospital without permanent complications. The patient was under constant control of both clinical and laboratory parameters. She was vaccinated using the Pfizer-BioNTech vaccine 12 months after total recovery in good health (two doses). During the six-week follow-up, no serious adverse reactions to the vaccine, changes in clinical status, or any abnormalities in laboratory tests were observed (Table 2). There was a significant increase in anti-SARS-CoV-2 IgG-specific antibody levels from 96.7 BAU (before vaccination) to >2080 BAU at 6 weeks post 2. dose of vaccine. The observed changes in laboratory results are presented in Table 2.

### 3.2. Case 2

The second presented patient is a 17-year-old male diagnosed with PIMS-TS in November 2020 and hospitalized at the Department of Pediatric Rheumatology. During the disease, he developed fever, conjunctivitis, cough, hypotension, pericardial effusion, and coagulation abnormalities. He had contact with his mother with COVID-19 four weeks before symptoms, but he did not develop symptomatic infection. 

Laboratory tests revealed high inflammation parameters (PCT, CRP, Il-6), in blood count: lymphopenia and thrombocytopenia, coagulopathy, hyperferritinemia, increased activity of transaminases, high level of troponin and N-terminal pro B-type natriuretic peptide (NT-proBNP) values. 

He was treated with IVIG and ASA. He was also discharged from the hospital ward with no abnormalities. In long-term observations, no symptoms relapsed. The patient received the Pfizer-BioNTech vaccine (2 doses) 9 months after total recovery and is in good health. During the six-month follow-up, nothing disturbing was observed, and the vaccine has proven safe and effective. There was a significant increase in anti-SARS-CoV-2 IgG-specific antibody levels from 58.7 BAU to >2080 BAU at 6 weeks post-vaccination (Table 3).

### 3.3. Case 3

Another patient is a 9-year-old female, diagnosed with PIMS-TS in April 2021, with no previously mentioned contact with a SARS-CoV-2 infected person, but a month earlier, she had suffered from mild pharyngitis. She presented fever, macular rash, gastrointestinal symptoms, conjunctivitis, pharyngitis, and coagulation abnormalities. Initially, she was treated with IVIG and ASA, but she developed macrophage activation syndrome (MAS). Therefore, the treatment was continued with GCS and cyclosporine. The girl was under constant clinical and laboratory observation and was vaccinated against COVID-19 nine months after PIMS-TS. She was vaccinated using the Pfizer-BioNTech vaccine 12 months after total recovery in good health (two doses). Six weeks after vaccination, no disturbing symptoms or laboratory indicators were observed for the second dose of the vaccine (Table 4).

Vaccination was effective via an increase in antibody levels from 167 BAU (before the vaccine to >2080 BAU (six weeks after the vaccine). 

### 3.4. Case 4

The 16-year-old male was diagnosed with PIMS-TS in January 2022. He was admitted to the hospital because of high fever, nausea, and vomiting, as well as abdominal and back pain. The patient had established contact with COVID-19 at school 4 weeks before the onset of the symptoms. He also received a second dose of the Pfizer-BioNTech vaccine 6 weeks before the onset of symptoms. During the hospitalization, a “raspberry” tongue, maculopapular rash, and ecchymosis appeared. The COVID-19 swab was performed twice, with both results being negative. Imaging studies showed free abdominal fluid, pleural effusion, lung consolidations, and enlarged paraaortic lymph nodes. Laboratory tests showed in blood count that leukocytosis, neutrophilia, and lymphopenia high levels of ESR, CRP, ferritin, D-dimers, LDH, and Il-6 increased transaminase activity (Table 5). After the treatment with IVIG, ASA, and GCS, his condition improved, and he was discharged in good health. However, as PIMS-TS was diagnosed post-vaccination, the vaccination course was stopped in his case. At the moment of admission, his serological status showed high levels of anti-SARS-CoV-2 IgG antibodies >2080 BAU (Table 5). Due to the confirmed contact with the SARS-CoV-2 positive patient, incomplete vaccination, and the lack of technical possibilities to determine antibodies against the N protein of the virus, it is not possible to determine whether the patient developed PIMS-TS in response to the vaccine or a viral antigen.

## 4. Discussion and Literature Review

The COVID-19 pandemic emerged as an unprecedented global health crisis, necessitating an urgent and coordinated scientific response to identify therapeutic interventions and preventive strategies. The rapid development and deployment of vaccines were central to these efforts, drawing on established principles of infectious disease control and immunology. Despite the demonstrable efficacy of vaccines in mitigating the morbidity and mortality associated with COVID-19, public discourse surrounding vaccination, particularly in the pediatric population, has been marred by skepticism and misinformation. Emerging clinical observations have confirmed that while children are generally at a lower risk of severe COVID-19, they are susceptible to specific post-infectious sequelae, notably Pediatric Inflammatory Multisystem Syndrome Temporally associated with SARS-CoV-2 (PIMS-TS) and the constellation of symptoms known as long-COVID. The protective efficacy of vaccines against severe COVID-19 manifestations in children is well documented; however, the extent to which vaccination influences the incidence or severity of PIMS-TS remains insufficiently characterized. Furthermore, the optimal timing of vaccination in the aftermath of PIMS-TS diagnosis is yet to be determined, and data on the potential of vaccines to precipitate PIMS-TS are sparse. This gap in knowledge underscores the imperative for rigorous scientific inquiry to elucidate the relationship between vaccination and these post-infectious conditions in the pediatric cohort. Such investigations are essential to inform evidence-based public health policies and vaccination strategies, thereby enhancing parental confidence in the safety and efficacy of COVID-19 vaccines for children. Comprehensive synthesis and analysis of the available data on these matters are critical for advancing our understanding and effectively communicating the benefits of pediatric vaccination against COVID-19, ultimately contributing to the mitigation of the pandemic’s impact on this vulnerable population. 

### 4.1. Effectiveness of Vaccination in Preventing the Development of PIMS-TS

Recent literature suggests a decline in the incidence of PIMS-TS during the latter stages of the COVID-19 pandemic, with vaccination playing a potentially significant role [11]. Studies highlight a correlation between vaccination rates and PIMS-TS cases, noting lower incidences in populations with higher vaccination coverage (Table 6). For instance, Holm et al. reported that among 11 PIMS-TS cases, 10 were unvaccinated [12]. Similarly, Zambrano et al. observed that 97 out of 102 eligible PIMS-TS patients had not received the vaccine, with none of the critically ill vaccinated children. Their findings suggest a 91% effectiveness of the Pfizer vaccine in preventing PIMS-TS [13]. Levy et al. noted that among 33 PIMS-TS patients, 7 had only received one vaccine dose, suggesting that SARS-CoV-2 infection occurred around the time of vaccination before full immunity could develop [14]. These findings are supported by unpublished data from our department, where no treated PIMS-TS cases had been vaccinated before their diagnosis, underscoring the potential protective effect of COVID-19 vaccination against PIMS-TS.

### 4.2. Vaccination Safety in Children Who Have Undergone PIMS-TS

The safety of vaccinating children post-PIMS-TS remains a critical question, given the known possibility of COVID-19 reinfections. While isolated cases hint at the complexity of post-PIMS-TS SARS-CoV-2 infection outcomes, broader studies suggest vaccination is both safe and effective for this group (Table 7). In the literature, there is the case of a male infected with the SARS-CoV-2 virus after undergoing PIMS-TS but did not develop symptoms of hyperinflammatory syndrome again and the case of a child with Down’s syndrome who probably relapsed with PIMS-TS [15,16]. In a paper published by Minoia et al., 15% of 236 centers taking part in the study confirmed SARS-CoV-2 reinfection after PIMS-TS, and two centers declared PIMS-TS flare [17]. In addition, the mere awareness that vaccination protects against the severe course of COVID-19 disease is sufficient to recommend this vaccination to this group of patients. For instance, Wisniewski et al. reported no adverse effects or PIMS-TS recurrence among 15 vaccinated PIMS-TS patients over 9.5 months [18]. Similarly, Aykac’s survey of 29 vaccinated post-PIMS-TS patients revealed reactions comparable to those in healthy children, with no significant issues or PIMS-TS relapse [19]. Hoste et al.’s international study of 273 children post-PIMS-TS also showed no concerning post-vaccination symptoms, with a single case of Bell’s paralysis resolving without complications [20]. A Spanish study involving 32 post-PIMS-TS patients vaccinated without severe adverse reactions or PIMS-TS recurrence further supports the safety of vaccination [21]. Enthralling data from MAS/sJIA and Vaccination Working Parties of the Pediatric Rheumatology European Society described the results of a survey involving respondents from over 60 countries who provide vaccinations to children with a history of PIMS-TS. The study showed that patients were vaccinated against COVID-19 only in 22 countries, but fortunately, there was good tolerance for vaccination and no serious adverse reactions [17].

### 4.3. Is It Possible to Develop PIMS-TS as a Result of the COVID-19 Vaccination?

The link between COVID-19 vaccination and the development of PIMS-TS remains uncertain due to the syndrome’s complex pathogenesis. Some reports suggest a potential association between vaccination and PIMS-TS onset, but deeper analysis often reveals these cases to be coincidental or influenced by pre-existing factors rather than directly caused by the vaccine (Table 8). For example, Jain et al. [22] and Collignon et al. [23] reported cases with a short interval between vaccination and PIMS-TS symptoms, suggesting other factors at play. Similarly, cases reported by Consollini et al. [24], Salzman et al. [25], and DeJong et al. [26] involved prior SARS-CoV-2 exposure, complicating the causal link to vaccination. In another case, Lee et al. described a child who developed PIMS-TS MIS-C after being exposed to COVID-19 despite the vaccination [27]. Santilli et al. reported PIMS-TS in two children with high somatic burdens shortly after COVID-19 vaccination, suggesting a potential link to an inadequate vaccine response [28]. Oudali et al. found 12 PIMS-TS cases among over 8000 patients, with 10 showing cardiac issues akin to post-vaccination myocarditis despite no prior SARS-CoV-2 contact [29]. Yalçinkaya et al. observed PIMS-TS in two individuals 27 days post-Moderna vaccine, with no anti-N antibodies detected [30]. Wangu et al. described a PIMS-TS case 10 weeks after the second vaccine dose, also without prior virus exposure [31]. Yousaf et al.’s analysis of over 21,000 vaccinated children revealed 8 PIMS-TS cases post-Pfizer vaccine with no SARS-CoV-2 contact history and 15 MIS-C cases post-vaccine with prior virus exposure, indicating a median onset of 34–35 days for PIMS-TS symptoms [32]. Varghese reported a potential PIMS-TS case in an 18-year-old 3 weeks after his third Pfizer dose, with no previous infection signs [33]. These cases suggest a possible, albeit rare, association between COVID-19 vaccination and PIMS-TS development, warranting further investigation. In many of the described cases, the presence of antibodies against the N protein of the virus was observed, which indicates previous contact with a person infected with the SARS-CoV-2 virus. In addition, a clinical study of the mRNA vaccine in a group of children 6-11 years of age showed no cases of PIMS-TS after vaccination [9]. Of course, this does not completely exclude the possibility that the vaccine becomes a trigger point in a predisposed person. However, such data argue against the possibility that vaccination by itself, via contact with the vaccine antigen alone, could cause PIMS-TS. In addition, in currently available literature, some cases have described children who have been diagnosed with type I diabetes, sickle cell anemia, or autoimmune diseases that may cause too weak antibody response. On the other hand, some authors managed to list extremely rare cases of developing PIMS-TS in temporal with vaccination and without prior contact with the virus. The course similar to myocarditis described as a complication after this vaccine indicates the need for meticulous and reliable reporting of changes in health status and extreme caution in assessing changes in the health status of children and considering PIMS-TS as a diagnosis despite (or because of) vaccination. In our case, it should be assumed that the boy developed PIMS-TS due to a combination of different factors rather than as a result of vaccination alone.

## 5. Conclusions

The analysis of the presented cases and literature data allows us to conclude that COVID-19 vaccinations in the group of patients with PIMS-TS are almost certainly as safe and effective in preventing PIMS-TS as in the group of healthy children [12,13,14,18,19,20,34,35,36]. Our data are consistent with the data provided by the previously mentioned authors. Moreover, the evaluation of the effectiveness measured as the increase in anti-SARS-CoV-2 antibodies indicated that the post-vaccination response in these patients is effective and undisturbed. In addition to our observations, studies show that mRNA vaccination against COVID-19 causes a more favorable immune response than being infected with COVID-19 or PIMS-TS [37,38]. Due to the data presented in the same study regarding the divergence in the approach to vaccination even within one country, it seems advisable to create guidelines that could be used by professionals to make the best decisions. Most often, patients were not vaccinated due to a sense of insufficient safety data and parental refusals. These data also indicate the need to monitor the health of patients after PIMS-TS but also to educate parents about the possible consequences of not vaccinating a child who is severely ill and often immunosuppressed. 

Most reported cases of post-vaccination PIMS-TS can be attributed to an incomplete response to the vaccine, both as a result of incomplete vaccination and the presence of immunosuppressive factors. Undoubtedly, cases of post-vaccination PIMS-TS in which there is no clear evidence of exposure to the virus should be closely monitored, and the vaccination cycle should be discontinued. Nevertheless, data on the effectiveness of at least a two-dose vaccination schedule in children, both in terms of disease prevention and PIMS-TS, strongly support the necessity of vaccination in this age group. 

A multifaceted approach is necessary to address the complex issue of childhood vaccination against COVID-19 and the potential risk of developing PIMS-TS. First, it is important to establish robust surveillance systems designed to monitor cases of PIMS-TS following vaccination of children against COVID-19. These efforts should be combined with targeted research initiatives to elucidate the incidence of this syndrome, its underlying mechanisms, and associated risk factors following vaccination. At the same time, it is of great importance to develop and disseminate clear, evidence-based guidelines for vaccination of children, especially those with a history of COVID-19 or PIMS-TS. These guidelines should include detailed recommendations on the optimal timing of vaccination for prior infection and recovery. Effective communication strategies also play a key role in this context, as they require transparent and accessible information on the risks and benefits of vaccinating children against COVID-19. It is important to address public concerns about potential side effects while emphasizing the key role of vaccination in preventing severe disease. Healthcare providers should be encouraged to take a personalized approach to vaccination decisions, taking into account each child’s individual health history, previous exposure to COVID-19, and specific risk factors for serious outcomes. Another key recommendation is post-vaccination monitoring for signs and symptoms of PIMS-TS, with healthcare workers being vigilant in the first weeks after vaccination. Establishing clear protocols for the management and treatment of suspected PIMS-TS cases will ensure timely and effective care. Finally, ongoing assessment of the safety of COVID-19 vaccines in children via post-marketing surveillance and research studies will provide valuable information on the risk-benefit profile of vaccines. As new evidence becomes available, vaccination guidelines and recommendations must be updated accordingly to reflect the latest scientific knowledge. By adopting this comprehensive approach, we can address the challenges of pediatric COVID-19 vaccination with care and precision, protecting children’s health and well-being while contributing to broader health goals.

## Figures and Tables

**Table 1:** Summary of PIMS-TS clinical manifestation in patients.

Case	Sex	Age	Symptoms	Treatment	Contact with Virus	Outcome
Case 1	Female	6	Fever, pharyngitis, macular rash, pericardial effusion and coagulation abnormalities	IVIG, ASA, GCS	3 weeks before symptoms; no symptoms of COVID-19 in the patient	Good
Case 2	Male	17	Fever, conjunctivitis, cough, hypotension, pericardial effusion and coagulation abnormalities	IVIG, ASA, GCS	Yes, no symptoms of COVID-19 in the patient	Good
Case 3	Female	9	Fever, macular rash, gastrointestinal symptoms, conjunctivitis, pharyngitis, coagulation abnormalities	IVIG, ASA, GCS, TOC, cyclosporine	No previously mentioned contact with SARS-CoV-2 infected person	MAS, finally good
Case 4	Male	16	High fever, abdominal and back pain, vomiting, nausea	IVIG, ASA, GCS	Contact 4 weeks before admission, after 2nd dose of the Pfizer-BioNTech vaccine.	Good

**Table 2:** Dynamics of changes in laboratory results and anti-SARS-CoV-2 IgG antibodies for Case 1.

Time	ESR [mm/h]	CRP [mg/L]	Ferritin [µg/L]	D-Dimer [ng FEU/mL]	WBC [10^3^/mm^3^]	LYM [10^3^/mm^3^]	Anti-SARS-CoV-2 IgG (BAU/mL)	Anti-SARS-CoV-2 IgM (BAU/mL)
Before treatment for PIMS-TS	76	7.65	503.90	2845.00	23.31	1.77	206.40	0.28
6 weeks after PIMS-TS	26	0.06	36.80	407.00	8.96	6.41	116.00	0.24
6 months after PIMS-TS	8	0.06	37.90	239.00	8.90	3.32	67.00	0.08
9 months after PIMS-TS	9	0.06	60.00	224.00	5.56	2.89	127.00	0.07
12 months after PIMS-TS (before vaccination)	6	0.06	41.20	160.00	5.33	3.02	96.70	0.00
6 weeks after 2nd dose of vaccine	15	0.18	32	340	13.80	3.61	2080.00	0.10

**Table 3:** Dynamics of changes in laboratory results and anti-SARS-CoV-2 IgG antibodies for Case 2.

Time	ESR [mm/h]	CRP [mg/L]	Ferritin [µg/L]	D-Dimer [ng FEU/mL]	WBC [10^3^/mm^3^]	LYM [10^3^/mm^3^]	Anti-SARS-CoV-2 IgG (BAU/mL)	Anti-SARS-CoV-2 IgM (BAU/mL)
Before treatment for PIMS-TS	62	9.45	303.90	3620.00	16.31	1.42	198.00	0.31
6 weeks after PIMS-TS	9.00	0.18	21.40	419.00	8.57	2.22	148.00	0.26
3 months after PIMS-TS	15.00	0.25	37.80	97.00	7.93	1.66	82.16	0.26
6 months after PIMS-TS	13.00	0.16	37.70	126.00	7.41	1.74	58.70	0.17
6 weeks after 2nd dose of vaccine	6.00	0.34	41.60	125.00	5.98	1.43	>2080.00	0.18

**Table 4:** Dynamics of changes in laboratory results and anti-SARS-CoV-2 IgG antibodies for Case 3.

Time	ESR [mm/h]	CRP [mg/L]	Ferritin [µg/L]	D-dimer [ng FEU/mL]	WBC [10^3^/mm^3^]	LYM [10^3^/mm^3^]	Anti-SARS-CoV-2 IgG (BAU/mL)	Anti-SARS-CoV-2 IgM (BAU/mL)
Before treatment for PIMS-TS	120.00	5.38	4367.00	7376.00	18.67	2.74	109.60	0.26
3 months after PIMS-TS	16.00	0.06	8.50	284.00	11.82	6.45	236.00	0.18
6months after PIMS-TS	11	0.06	18	324,00	8.43	4.29	167.00	0.20
6 weeks after 2nd dose vaccine	9	0.06	22	256,00	7.56	3.73	>2080	0.22

**Table 5:** Dynamics of changes in laboratory results and anti-SARS-CoV-2 IgG antibodies for Case 4.

Time	ESR [mm/h]	CRP [mg/L]	Ferritin [µg/L]	D-dimer [ng FEU/mL]	WBC [10^3^/mm^3^]	LYM [10^3^/mm^3^]	Anti-SARS-CoV-2 IgG (BAU/mL)	Anti-SARS-CoV-2 IgM (BAU/mL)
Before PIMS-TS treatment	54.00	25.27	598.30	4273.00	16.98	0.64	>2080	0.32
6 weeks after PMS-TS	7	0.06	114.00	464.00	12.56	1.68	>2080	0.24

**Table 6:** Effectiveness of COVID-19 vaccines against PIMS-TS.

Study	Group	Effectiveness	Comments	References
Holm, M. et al.	11 children were diagnosed with PIMS-TS between January and March 2022.	10/11 of the children were unvaccinated.	Data from all pediatrics departments in Denmark.	[12]
Zambrano et al.	102 PIMS-TS patients, aged from 12–18 (qualified for vaccination)	97/102 of the children were unvaccinated, and the whole group with the life-threatening outcome (38 patients) was unvaccinated.	Data from 24 pediatric departments in 20 US states.	[13]
Levy, M. et al.	33 children with PIMS-TS who were eligible for vaccination.	Of the 33 children with PIMS-TS who were eligible for vaccination, none were fully vaccinated, and 7 received only one dose.	The median time between receiving one dose of vaccine and MIS-C was 25 days.	[14]

**Table 7:** Safety and efficacy of COVID-19 vaccination after PIMS-TS.

Paper	Group	Interval between MIS-C and Vaccination	Results	References
Wisniewski, M. et al.	15 vaccinated patients after recovery from PIMS-TS	91–341 days	No adverse effects or relapse of PIMS-TS were observed within 9.5 months of vaccination.	[18]
Aykac, K. et al.	29 vaccinated patients after recovery from PIMS-TS	Max. 208	No statistically significant differences in adverse reactions between groups of children with PIMS-TS compared to healthy children.	[19]
Hoste, L. et al.	273 children from 32 countries	no data	No adverse effects (despite one child with Bell’s palsy) or relapse of PIMS-TS were observed in all of the patients.	[20]
Epalza C, et al.	32 vaccinated patients with previous PIMS-TS in the Spanish population	Median 42 weeks	No severe adverse events, no PIMS-TS relapse, no myocarditis.	[21]
Minoia F, et al.	Data from 236 centers from 61 counties.	3–6 months (40%), 6–12 months (52%), or >12 months (8%)	Only six reported complications, among them 3 mild symptoms and one PIMS-TS-like reaction.	[17]

**Table 8:** PIMS-TS cases after COVID-19 vaccination.

Paper	Case	The Interval between Vaccination and MIS-C	Comments	References
Aykac, K. et al.	3 cases of PIMS-TS after SARS-CoV-2 vaccination	4–13 days	In one case, positive PCR.	[19]
Jain, E. et al.	Two PIMS-TS cases following Pfizer BioNTech vaccine	<1 week	One case with positive for anti-N but negative for anti-S SARS-CoV-2 protein.	[22]
Collignon, C. et al.	1st case: PIMS-TS four days after 1st dose of Pfizer BioNTech 2nd case: PIMS-TS three days after 1st dose of Pfizer BioNTech	4 days and 3 days	1st case—diabetes type I, SARS-CoV-2 infection 7 months earlier 2nd case—positive anti-N IgG antibodies	[23]
Consolini R, et al.	Case of a 17-year-old vaccinated girl, she developed PIMS-TS despite two doses of mRNA vaccine.	5–6 months	10 days before admission, contact with SARS-CoV-2 positive person.	[24]
Salzman M. et al.	Two PIMS-TS cases following the Pfizer BioNTech vaccine	15 days and 19 days	Two out of three cases meet age criteria. One patient with positive swabs and anti-N antibodies. The second patient reported COVID-19 infection 6 weeks later, positive for anti-N antibodies.	[25]
DeJong, J. et al.	Case of PIMS-TS following two doses of the Pfizer BioNTech vaccine.	2 months	Girl diagnosed with Sickle Cell Disease. Positive for anti-N antibodies.	[26]
Lee, S. et al.	Case of PIMS-TS after vaccination (13 weeks before PIMS-TS) and COVID-19 infection (6 weeks before PIMS-TS)	13 weeks.	Reported infection.	[27]
Santilli, V. et al.	2 cases of PIMS-TS after SARS-CoV-2 vaccination presented with neurological symptoms	1–10 days	Positive anti-N antibodies, autoimmunity, and reported contact with COVID-19.	[28]
Ouldali, N. et al.	8 suspected PIMS-TS cases following mRNA anti-SARS-CoV-2 vaccine.		In all 8 cases, past infection with SARS-CoV-2 has been excluded. Data was obtained from 8,113,058 people.	[29]
Yalçinkaya, R. et al.	Case of PIMS-TS following one dose of Pfizer BioNTech vaccine.	27 days	Negative for anti-N antibodies.	[30]
Wangu, Z. et al.	Case of PIMS-TS following the second dose of the Pfizer BioNTech vaccine.	10 weeks	Negative PCR swab and negative for anti-N antibodies.	[31]
Yousaf, A. et al.	8 cases of PIMS-TS following at least one dose of Pfizer BioNTech vaccine with no history of contact with SARS-CoV-2 and 15 cases of PIMS-TS following at least one dose of Pfizer BioNTech vaccine with a history of contact with SARS-CoV-2.	Median 34 and 35 days	The whole analyzed population included 21 335 331 vaccinated children from the USA.	[32]
Varghese, M. et al.	Case of an 18-year-old male with MIS-C diagnosed after a booster dose of the Pfizer BioNTech vaccine.	3 weeks	Negative anti-N antibodies in IgG and IgM classes as well as PCR swabs.	[33]

## Data Availability

Data are available upon written request from the first author of this manuscript.
